# Case Report: Cariprazine in a Patient With Schizophrenia, Substance Abuse, and Cognitive Dysfunction

**DOI:** 10.3389/fpsyt.2021.727666

**Published:** 2021-08-18

**Authors:** Jose Rodriguez Cruz, Johan Sahlsten Schölin, Stephan Hjorth

**Affiliations:** ^1^Department of Psychosis, Sahlgrenska University Hospital, Mölndal, Sweden; ^2^Pharmacilitator AB, Vallda, Sweden; ^3^Department of Molecular and Clinical Medicine, Institute of Medicine, The Sahlgrenska Academy at Gothenburg University, Gothenburg, Sweden

**Keywords:** cariprazine, quetiapine, antipsychotics, drug abuse, cognitive dysfunction, negative symptoms schizophrenia

## Abstract

This case report describes a 30-year old male diagnosed with schizophrenia at the age of 23, and with a long history of drug abuse. He had previously received a wide range of antipsychotic drug treatment regimens, all with some degree of effect, but never with complete symptom relief. He was also suffering from persistent cognitive and negative symptoms. At the time of admission in our clinic, he was on Quetiapine (QUE) and Haloperidol (HAL). It was therefore decided to substitute HAL for Cariprazine (CAR)—an agent with a novel pharmacological and clinical profile—in the hope of gaining increased efficacy, particularly in the cognitive and negative symptom domains. Within 3 weeks of the switch from HAL to CAR the patient clearly improved, and notably so in the aforementioned symptom areas. A number of subsequent adjustments of antipsychotic dosages and adjunct medications during the ensuing months resulted in an apparently more stable alleviation of positive as well as negative and cognitive symptoms, including markedly improved personal and social capabilities. Interestingly, some time after initiating CAR treatment the patient also reported that from being a heavy smoker (60 cig/d) he had cut down and eventually ceased smoking entirely; furthermore, he has remained clean of other substance abuse since his first admission in 2020. The joint treatment with CAR in combination with QUE thus seems to have improved the patient's cognitive functioning as well as possibly his susceptibility to substance abuse.

## Introduction

Schizophrenia is a chronic disorder with variable clinical features and changes in numerous aspects of mental processing. It causes significant and long-lasting impairments, makes heavy demands for hospital care and requires extensive efforts from the healthcare system and other actors. Substance abuse is also common in individuals with schizophrenia, the association of which links to symptom exacerbation, poorer medication compliance, deterioration of functioning, higher risk of hospitalization and overall increased costs to the individual and society (1–3). In this regard, very frequent drug comorbidities are marijuana, and psychostimulant agents like amphetamine and cocaine (1, 4).

Cognitive impairments are also common in patients suffering from schizophrenia, especially in the domains of attention, executive function and memory. As these are considered a better predictor of level of function in outpatients than is the severity of psychotic symptoms, cognitive impairments have become the target of many pharmacological and psychosocial treatment trials (5).

Cariprazine (CAR) is a third-generation antipsychotic, approved in Europe 2017 for the treatment of schizophrenia. Several studies suggest that CAR may be of particular interest with regard to treatment of negative and cognitive symptoms in patients with schizophrenia (6–8). Intriguingly, some reports additionally suggest its usefulness to improve symptoms of substance use disorder (9, 10), although prospective randomized studies are necessary to evaluate this further.

We present a case of a patient with concomitant schizophrenia and substance abuse disorder admitted to an in-patient psychiatric unit due to increasing psychotic symptoms. He had previously been treated with a wide range of antipsychotic drugs. While all of these regimens had had some effect, none had led to complete symptom remission and the patient was suffering from persistent negative and cognitive symptom manifestations.

## Case Presentation

A 30-year old male was admitted to an in-patient psychiatric unit for treatment, after at least 3 weeks of escalating psychotic symptoms, consumption of amphetamine and treatment compliance failure (Quetiapine 900 mg/d, Lithium 168 mg/d, Mirtazapine 45 mg/d). The months prior to the hospitalization there was a slow decline in function [described in detail under section Current episode (overview in **Table 2**), below] and increase in psychotic symptoms seen correlating with social stressors, and finally with drug intake.

### Background History

He reached 12th grade of education (~senior high school). Since childhood this patient had shown difficulties in his social interactions. According to caregiver interviews, there were premorbid symptoms, including early behavioral and social deviation. At the age of 18 he started consuming cannabis daily, with breaks no longer than a month. He intermittently used alcohol, opioids, LSD, and benzodiazepines but he tended to prefer cannabis and psychostimulants such as amphetamines.

The first psychotic episode was seen in proximity to drug intake. At this time, he was 23 years old, he had been using cannabis and amphetamines daily during 5 and 3 years, respectively, and sought healthcare for affective symptoms: depression and anxiety. His age of onset of psychosis is thus within the typical range for schizophrenia debut. As he was already advanced in his disease, investigations were made from a neuropsychiatric perspective, and a clinical picture of Attention Deficit Disorder (ADD) and Autism-Spectrum Disorder (ASD) was recognized.

He was described as easily distractible and sensitive to stress. Negative symptoms were also a central source of his general dysfunction. His way of interacting and the lower than expected level of self-care often had a quality that could be mistaken for autism. He expressed satisfaction in taking part in social/rehabilitation training/work, but he had a limited capacity to absorb instruction/new information.

Prior to the current admission the patient had had several admissions to psychiatry inpatient units. His first episode at the age of 23 was deemed to be an amphetamine-induced psychosis, but the diagnosis was later reconsidered and re-labeled schizophrenia. At that time the patient was suffering terrifying auditory hallucinations and religious delusions, even intolerable anxiety and fear that caused self-harm. He had occasional psychomotor agitation and aggressive behavior but mainly marked motor inhibition, inhibited facial expression, alogia and flattened affect, anhedonia, hypobulia, and isolation. He suffered extended periods of catatonia.

Over a period of ~7 years prior to the present admission he had been prescribed several different antipsychotic treatments with good compliance, but simultaneously continued to consume drugs and got variable efficacy and side effects. He showed persistent psychosis symptoms despite periods of abstaining from dependence-producing agents that could extend to 8 months; this according to the patient's own information, since current urine drug screen methods (UTox) may not catch all types of illicit agents (e.g., “spice,” and net-drug variants). Table 1 summarizes previous treatment regimens.

**Table 1:** Medication history overview (in- as well as outpatient periods).

**Agent**	**Dose/s**	**Period**	**Comment**
Haloperidol (HAL)	Up to 10 mg/d	2015 (4 mths) 2018 (1 mth) 2020 (2 wks)^*^	Combined with diazepam improved the most distressing hallucinations but caused intolerable extrapyramidal side effects (EPS).
Risperidone (RIS)	6 mg/d	2015–2016 (~1 year)	Partially relieved the positive symptoms despite suspected concomitant drug use, but caused EPS and ejaculatory dysfunction. It was replaced by Clozapine, and after a second attempt (4 mths in 2017 during a later admission event) by Quetiapine.
Olanzapine (OLA)	Up to 30 mg/d prn 7.5–15 mg during exacerbations	2018 (2 mths)	Good sedative effect and transiently improved hallucinations and delusions.
Clozapine	Up to 700 mg/d	2015–2018 (~3 yrs)	The most effective medication according to the medical staff. The patient reported asymptomatic periods alternating with periods of controllable symptoms, though never a permanent relief.
Quetiapine (QUE)	900 mg/d	Current	The most effective medication according to the patient, particularly against hallucinations and anxiety, but still without a permanent relief as monotherapy.
Aripiprazole	Up to 30 mg/d	2017 (3 wks, October)	The patient refused to continue with it and stated that “it was not good” for him, without further explanation.
Lithium	Up to 210 mg/d	2018 (October) - current	Used as a calming agent.

* *Discussed in the current report*.

Benzodiazepines (Diazepam, Clonazepam, Oxazepam, or Lorazepam) as well as antihistamine compounds (Levomepromazine and Alimemazine) were periodically used for the treatment of anxiety, self-harm and agitation in addition to the antipsychotics. Electroconvulsive therapy (ECT) was effective during prior episodes of catatonia.

### Current Episode

An overview of events, medications, and associated comments is found in Table 2. By the time of our contact in June 2020, at the age of 30, this patient also used drugs, mainly marijuana and amphetamines. He showed cognitive dysfunction (attention, working memory, cognitive flexibility and spontaneity), as well as worsened social skills and emotional responses that had previously been interpreted as ASD. He also complained about facial tics and tremors. As evident from the above, our patient had previously been treated with a wide range of antipsychotic drugs, none of which was more than partly effective. He was also suffering from persistent cognitive and negative symptoms.

**Table 2:** Timeline summary of patient events and medication across the current admission history.

**Date**	**Event**	**Medication/s**	**Comment**
Wk 23, 2020	**ADMISSION**	QUE (900 mg/d), Lithium (168 mg/d), Mirtazapine (45 mg/d)	Severe positive and negative symptoms Cognitive dysfunction and social/emotional impairment
Wk 27, 2020	Psychotic worsening, agitation, disorganized behavior	RIS (6 mg/d) add-on	
Wk 28, 2020		Switch from RIS to HAL (7,5 mg/d)	Non-compliance oral RIS → intramuscular HAL
Wk 30, 2020	Decision to switch to from HAL to CAR	QUE (900 mg/d) + HAL (7.5 mg/d) Start CAR (1.5 mg/d → 6 mg/d @ d9) Begin taper HAL on d9 of CAR	
Wk 32, 2020		QUE (900 mg/d) + CAR (6 mg/d)	Alleviation of paranoid delusions and auditory hallucinations - marked improvement in negative symptoms
Wk 33, 2020	End tapering of HAL	QUE (900 mg/d) + CAR (6 mg/d)	Sudden significant improvement in social interaction, self-care
Wk 33, 2020	EPS and akathisia	QUE (900 mg/d) + CAR (6 mg/d) Reduction of CAR dose (to 4.5 mg/d) Add-on w Propranolol (90 mg/d) + Biperiden (4 mg/d)	
Wk 34, 2020	Start down-titrating QUE to 300 mg/d	QUE (900 mg/d) + CAR (4.5 mg/d) Antihistamines and benzodiazepines available prn for possible QUE rebound symptoms	Antihistamines and benzodiazepines available prn
Wk 40, 2020	Patient **DISCHARGE**	QUE (300 mg/d) + CAR (4.5 mg/d)	Plasma CAR_tot_ in expected therapeutic range Start part-time work rehabilitation
Wk 41-42, 2020	Return of auditory hallucinations	QUE (300 mg/d) + CAR (4.5 mg/d)	
Wk 43, 2020	Persistent auditory hallucinations	QUE (300 mg/d) + CAR (4.5 mg/d) Add-on w OLA (10–20 mg/d)	
Wk 44, 2020	Add-on w OLA, increase CAR (4.5 → 6 mg/d)	QUE (300 mg/d) + CAR (4.5 mg/d) Add-on w OLA (10–20 mg/d)	
Wk 45, 2020	Persistent auditory hallucinations → **READMISSION**	QUE (300 mg/d) + CAR (6 mg/d) Add-on w OLA (10–20 mg/d)	Retained emotional/social improvement, partial disease insight vs. 6 wks earlier; marked reduction of tobacco use
Wk 46, 2020	Return to previously successful treatment regime	QUE (300 mg/d) + CAR (6 mg/d) QUE up-titration and OLA down-titration	
Wk 47, 2020	Voice-evaluation scale scoring	QUE (300 mg/d) + CAR (6 mg/d) QUE up-titration and OLA down-titration	Auditory hallucinations alleviated
Wk 48, 2020	Reduce CAR dose Medication adjustment done	QUE (900 mg/d) + CAR (4,5 mg/d)	Stabilized positive symptoms, but suffering from akathisia and anxiety
Wk 49, 2020	Patient **DISCHARGE**	QUE (900 mg/d) + CAR (4.5 mg/d) Add-on w Clonazepam (2 mg/d) + still on Propranolol	Returned home, restarted work rehabilitation (2 h, 3 times/wk), started going to gym
Wk 9, 2021		QUE (900 mg/d) + CAR (4.5 mg/d) Add-on w Clonazepam (2 mg/d) + still on Propranolol	Quit smoking
Wk 11, 2021		QUE (900 mg/d) + CAR (4.5 mg/d) Add-on w Clonazepam (2 mg/d) + still on Propranolol	Increased work rehabilitation to 10 h/wk; shortly thereafter reported stress and voices
Wk 13, 2021		QUE (900 mg/d) + CAR (4.5 mg/d) Add-on w Clonazepam (2 mg/d) + still on Propranolol	Returned to 6 h/wk work rehabilitation scheme; no drugs of abuse found in regular testing since last discharge

After weeks (possibly months) of progressive deterioration with increasing paranoia and hallucinations he was subject to forced psychiatric clinical admission. At this time there had for 2–3 months been a looming notice of losing his apartment, and there was a suspicion of probable compliance failure. During the last house call, he wore dirty clothes, was verbally aggressive, openly hallucinating and expressed feeling threatened to his life by the medical staff. He showed alogia and aggressive speech with flattened affect. The patient had no disease insight.

In the inpatient unit he was reinstated on previous treatment and dosages: QUE (900 mg/d), Lithium (168 mg/d) and Mirtazapine (45 mg/d). On week 4 he showed worsening symptoms of psychosis, including bursts of agitation and violence toward his environment and himself, as well as disorganized behavior, e.g., collecting garbage in his room. It was handled with an add-on with RIS (6 mg/d). On week 5 we had to switch from RIS to HAL, to afford an intramuscular alternative due to non-compliance with oral administration.

On week 7 after admission, the decision was taken to switch from HAL to CAR (with a target dose of 6 mg/d on day 9). At the start of this switch his HAL dose was 7.5 mg/d and QUE 900 mg/d. Tapering of HAL was initiated on day 9 after starting CAR. HAL was down-titrated and stopped over 2 weeks. The patient responded well both in terms of alleviation of auditory hallucinations and paranoid delusions, he also markedly improved in negative symptoms. Coinciding with the expected time for 90% of steady-state levels of CAR [~3 weeks; (11)], he rather suddenly started to pay attention to his personal care and appearance and displayed a significant positive shift in the quality of his social interactions. The patient improved in terms of eye-contact, conveying more spontaneous and meaningful speech as well as a progressive development of self-reflecting capacity.

Three weeks after the start of CAR the patient complained of EPS and akathisia. The dose was therefore reduced 4.5 mg/d and Propranolol 90 mg/d and Biperiden 4 mg/d concurrently added. Four weeks into the treatment with CAR we started down-titrating QUE to 300 mg, applying a plateau-switch strategy (12). During this time, he had access to both antihistamines and benzodiazepines to alleviate possible rebound issues.

He was discharged on week 10 after admission with significant improvements in both negative and positive symptoms. At this time the concentration of total CAR was 84 nmol/L (S-CAR 10 + S-Desmethyl-CAR 4 + S-Di-desmethyl-CAR 70 nmol/L), which is well within the expected range [20–150 nmol/L with doses 1.5–6 mg/d; (11)].

At home he returned to work rehabilitation and leisure activities, like playing the guitar. Auditory hallucinations returned 1–2 weeks after discharge in September 2020. A week later the patient was given an add-on with OLA 10–20 mg/d. The following week his dose of CAR was increased from 4.5 to 6 mg. His QUE was still at 300 mg. However, the symptoms did not abate, and he was readmitted.

It was noted that he retained emotional contact qualities, and at least partial disease insight as compared to prior the initiation of CAR 6 weeks earlier (July 2020). As the previous combination of QUE 900 mg/d and CAR 4.5–6 mg/d apparently was used successfully during the last hospitalization, our first step was to return to that regime.

OLA was down-titrated and eventually stopped completely on day 14, while QUE was increased to reach a full dose of 900 mg/d on day 14. He slowly stabilized regarding his positive symptoms but was suffering from akathisia and anxiety. These symptoms appeared closely related and both were therefore managed by decreasing the dose of CAR from 6 to 4.5 mg/d and adding Clonazepam 2 mg/d (the patient was still on beta-blockade).

His auditory hallucination experiences were evaluated with the use of a “Voice-evaluation scale” (13), 2 days after his release from 10 weeks of in-patient care (June–September 2020) and 2 weeks after readmission October 2020. He scored the same total points both times using this assessment instrument. At the time of discharge on 4th of November, after 4 weeks of in-patient care, the auditory hallucinations are described as much attenuated, lesser in frequency and intensity. Overall the patient describes heightened well-being and plans for the future.

After another 4 weeks he was discharged and returned to work rehabilitation. The subsequent 2 months the patient attended his work-training 2 h, 3 times/week. He reported finding it stimulating and enjoyed being around other people. He started visiting the gym frequently. He increased his work-training attendance to 10 h a week. Shortly after this increase he reported a sense of stress and increased voices. Two weeks later he returned to the previous 6 h per week schedule (but the possibility of increasing the number of hours daily is kept open).

The patient was at this time regularly tested for any substance use but remained clean of such since the first hospitalization in 2020 (verified by regular UTox testing). When he returned for the second inpatient period in October 2020, a month after discharge, it was also noticed that he had spontaneously cut down on tobacco use. He had been a regular smoker since the age of 18 (now 30) with a consumption of about 60 cigarettes per day. Two months after the end of his second inpatient stay, he quit smoking cigarettes entirely and now uses only tobacco- and nicotine-free e-cigarettes.

According to the UKU-scale (“Udvalg for Kliniske Undersøgelser” Side Effect Rating Scale) February 2021, 6 months after the first discharge, there is an improvement as compared to half a year ago (September 2020) with respect to quality of sleep, less emotional numbness, less EPS (stiffness, myalgia and bradykinesia). He has an easier time sitting still and relaxing. No more facial tics are evident, and there is less tremor of the hands.

## Discussion

As is often the case, there aren't clear cut diagnostic features for a patient with longstanding psychosis. In the current account, the neuropsychological evaluation was made in adult age, and only after a while it became known that the patient had engaged in frequent recreational substance abuse on and off from late adolescence. The possible contributory aspects from drug consumption makes the precision of a neuropsychiatric diagnosis less reliable. We thus believe that besides the obvious and independent positive symptoms of schizophrenia, the overall profile in this case should be interpreted as a combination of schizophrenia with its premorbid symptoms and negative symptoms, as well as superimposed harmful effects of substance abuse. It is difficult to say whether our patient would have developed a primary psychosis in the absence of substance use, but it seems certain that substance use relapses have driven the relapse in psychosis more than once since his debut. It has also been a hindrance for effective treatment.

At the time when it was decided to add CAR to our patient's treatment he had previously been treated with a wide range of antipsychotic drugs. All of these had shown some effect, but none had led to complete symptom relief, and the suffering from negative and cognitive symptoms persisted. For example, the D2 receptor blocking agents (RIS or HAL) previously used to handle intense positive symptoms were not satisfactory, neither from a negative and cognitive symptom nor a side effect perspective. A Clozapine treatment course earlier in his illness history had been accompanied with some effect also on negative symptoms, but we speculate that this was related to increased abuse of stimulating substances during this period in time. We thus decided to try QUE as an add-on, an agent known to be pharmacologically different and with less D2 receptor impact than the former two high-affinity antagonists. The choice of antipsychotic agent in this case was also based on the express notion that trying a medicine from a different antipsychotic drug class should be attempted if the prior compound has not had the desired effect (5, 12).

The history of severe positive symptoms as well as clear cognitive and negative dysfunction, indicated to us that our patient needed strong support to improve all of these issues. Accordingly, and with the lack of any marked success with previous treatment regimens in mind, our ambition was to test an antipsychotic drug not previously tried; this in order to simultaneously address his positive as well as the prominent cognitive and negative symptoms. The basic pharmacological and clinical profile of CAR points to significant beneficial impact in particular with regard to the negative and cognitive symptom domains, in addition to its efficacy *vs*. positive symptoms (6–8, 14, 15). CAR therefore appeared to provide us with an option with a particularly good fit regarding our patient's symptom expressions, and hence worthwhile to try.

When CAR treatment was initiated he was on a regimen of QUE (900 mg/d) and HAL (7.5 mg/d). The latter agent was used during the intense acute phase of the hospitalization. According to the deliberations above, we choose to switch from HAL to CAR, applying a relatively fast up-titration of the latter, reaching 6 mg/d in 7 days; QUE was retained throughout. HAL was tapered beginning on the 8th day after initiating CAR, and an alleviation of both negative and positive symptoms were evident 2–3 weeks after starting CAR. Incidentally, this time-scale coincides with the predicted reach of steady-state levels of CAR and its main active metabolite [di-desmethyl-CAR; (11)]. The patient then exhibited a sense of increased awareness of disease that could even be described as an increase of insight. Similar changes had been seen before with this patient and it is hard to tell whether it was a new development or an even deeper insight than earlier. Regardless, clinically he engaged clearly more collaboratively around his treatment than the typical schizophrenia patient who needs to be persuaded to agree to treatment. The impression thus was that the effect of the CAR + QUE combination treatment was superior to any of his previous medication trials. During late 2020, adjustments of the doses of both agents were done to find the optimal regimen for our patient, eventually ending in CAR (4.5 mg/d) and QUE (900 mg/d) that appeared to be the best choice for him (see, Table 2).

To add to the overall equation, our patient had previously had repeated relapses that seemed at least partly related to substance use. He had tried a number of antipsychotic drugs before, all moderately effective but never leading to complete remission; at some point, even accompanied by relapse in substance use. We therefore speculate that he may have used classified drugs at the end of a deteriorating course in his disorder as a “self-medication” substitute to healthier, better functioning coping strategies. Notably, 2 months after initiating CAR treatment, our patient spontaneously reported that he had decreased cigarette smoking (from 60 cigarettes/d). About 5 months later he stopped altogether and now instead uses e-cigarettes. Interestingly, during this period, all of the urine screen tests (UTox) run for illicit drugs in this patient were negative. We believe that if the CAR treatment contributed to help him stay off substances it may increase his chances of continuous remission. Preclinical theory and findings along with recent clinical case reports are consistent with the idea that partial D3 receptor agonism by CAR may indeed be helpful in the treatment of drug dependence conditions (9, 10, 16–18). In a best-case scenario, his concomitant sudden and unexpected smoking cessation and lack of indication of continuing use of recreational substances could signify a dampened drive for substances of abuse. If so, CAR may increase his chances of continuously abstaining from drugs, and by doing so also improve his chances to sustain prolonged remission. In support of this speculation, a recent paper reported the successful remission from persistent methamphetamine psychosis by CAR treatment (19). Interestingly, in another study the abuse of alcohol and cannabis in three bipolar I patients was also attenuated following treatment with CAR—one case of which in fact achieved markedly reduced alcohol craving and sustained stability upon combined CAR and QUE treatment (10).

**Figure 1:**
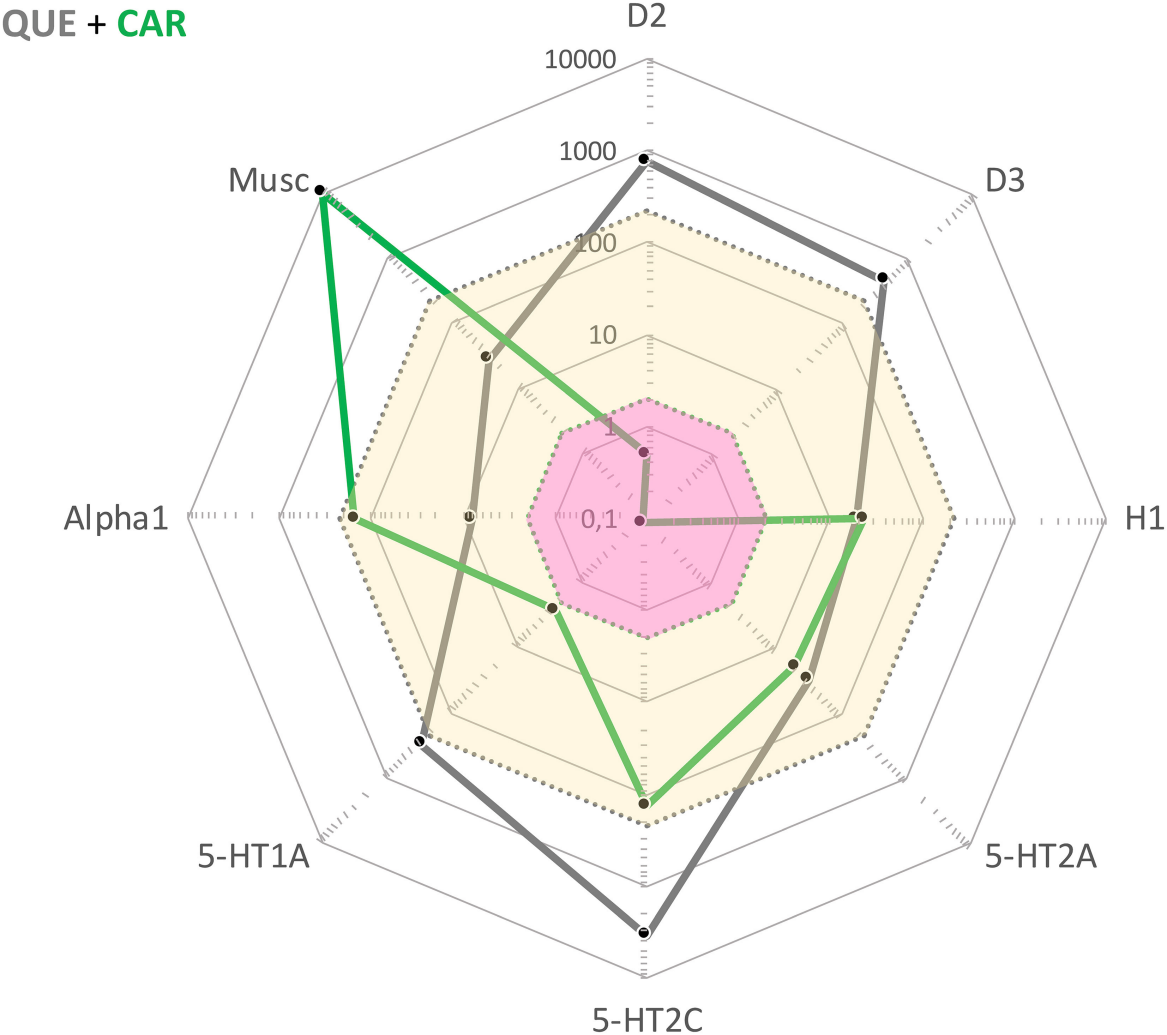
Cobweb depiction of Quetiapine (QUE) and Cariprazine (CAR) target profiles overlaid on the free steady-state plasma concentrations of these antipsychotics at average clinical dosage (QUE: yellow area; CAR: pink area). Black dots correspond to drug affinities reported in the literature (in nM) for the targets labeled on the edges of the cobweb; the closer to the center, the higher affinity for the target in question.

In summary, for this particular patient the most effective treatment to date was a combination of QUE and CAR. Both of these drugs had been tried and deemed insufficient in monotherapy, but apparently synergized with regard to clinical efficacy once combined. To the best of our knowledge, this is not a treatment combination specifically recommended in any guidelines. This said, the complementary drug target profiles make sense from a pharmacodynamic perspective (see, Figure 1). While QUE shows rather poor D2 receptor antagonist properties along with intermediate affinities for, i.a., histamine H1 and 5-HT2A receptor sites, CAR has very high affinity and partial agonist properties at D3, D2, and 5-HT1A receptor sites. Furthermore, the combination of one antipsychotic with short (QUE) and one with extended half-life (CAR) is potentially favorable from a compliance point-of-view as it may provide a “buffering” capacity *vs*. therapeutic target occupancy fluctuation. This complementarity in pharmacodynamic, and also pharmacokinetic, features may tentatively underlie the beneficial clinical outcome in the current patient case.

### Strengths and Limitations

Needless to say, a limitation of the work is that it is based on the description of a single patient case, thus limiting generalizations to a wider patient population. However, the comprehensive and detailed account of the diagnosis and close management follow-up from the clinical and pharmacological perspective is a clearcut strength, particularly as one of the authors (JRC) has been able to follow the disease and treatment course of this patient over several years.

## Conclusion

It seems that CAR add-on to QUE treatment improved cognitive functioning and desire for addictive substance use in our patient. From an antipsychotic polypharmacy perspective, the CAR + QUE combination also appears to provide a pharmacodynamically as well as pharmacokinetically attractive treatment option with complementarity across several clinically relevant medication aspects. Prospective randomized studies are necessary to extrapolate the predictability of our findings to the broader population of individuals with schizophrenia, including patients using addictive substances.

## Data Availability Statement

The datasets presented in this article are not readily available because the data are extracted from a patient medical journal, and is thus personally confidential within the framework of the medical professionals involved in his treatment. Requests to access the datasets should be directed to José Rodriguez Cruz, jose.rodriguez_cruz@vgregion.se.

## Ethics Statement

Ethical review and approval was not required for the study on human participants in accordance with the local legislation and institutional requirements. The patients/participants provided their written informed consent to participate in this study. Written informed consent was obtained from the individual(s) for the publication of any potentially identifiable images or data included in this article.

## Author Contributions

JR and JS were in charge of the patient's clinical management and wrote the original draft. JR, JS, and SH conceptualized and researched the subject, conceptualized, reviewed, and edited the manuscript. All authors contributed to the article and approved the submitted version.

## Conflict of Interest

The authors have received honoraria for scientific talks and participation in advisory boards from Recordati. The writing of this report was in part sponsored by Recordati, but the company had no influence on data collection, analysis, content, or interpretations. None of the authors holds any shares in the company.

## Publisher's Note

All claims expressed in this article are solely those of the authors and do not necessarily represent those of their affiliated organizations, or those of the publisher, the editors and the reviewers. Any product that may be evaluated in this article, or claim that may be made by its manufacturer, is not guaranteed or endorsed by the publisher.

## Abbreviations

UKU: Udvalg før Kliniske Undersøgelser
CAR: Cariprazine
QUE: Quetiapine
HAL: Haloperidol
OLA: Olanzapine
EPS: Extrapyramidal Side Effects.
